# Applications of Hyaluronic Acid in Ophthalmology and Contact Lenses

**DOI:** 10.3390/molecules26092485

**Published:** 2021-04-24

**Authors:** Wan-Hsin Chang, Pei-Yi Liu, Min-Hsuan Lin, Chien-Ju Lu, Hsuan-Yi Chou, Chih-Yu Nian, Yuan-Ting Jiang, Yuan-Hao Howard Hsu

**Affiliations:** 1 Research and Development Center, Yung Sheng Optical Company, Daya District, Taichung 42881, Taiwan; wanhsinchang@hydron.com.tw (W.-H.C.); ginaliu@hydron.com.tw (P.-Y.L.); hsuanlin@hydron.com.tw (M.-H.L.); rurulu@hydron.com.tw (C.-J.L.); nu5544@hydron.com.tw (H.-Y.C.); joseynian@hydron.com.tw (C.-Y.N.); 2 Department of Chemistry, Tunghai University, Xitun District, Taichung 40704, Taiwan

**Keywords:** hyaluronic acid, contact lenses, ophthalmology

## Abstract

Hyaluronic acid (HA) is a glycosaminoglycan that was first isolated and identified from the vitreous body of a bull’s eye. HA is ubiquitous in the soft connective tissues of animals and therefore has high tissue compatibility for use in medication. Because of HA’s biological safety and water retention properties, it has many ophthalmology-related applications, such as in intravitreal injection, dry eye treatment, and contact lenses. Due to its broad range of applications, the identification and quantification of HA is a critical topic. This review article discusses current methods for analyzing HA. Contact lenses have become a widely used medical device, with HA commonly used as an additive to their production material, surface coating, and multipurpose solution. HA molecules on contact lenses retain moisture and increase the wearer’s comfort. HA absorbed by contact lenses can also gradually release to the anterior segment of the eyes to treat dry eye. This review discusses applications of HA in ophthalmology.

## 1. Introduction

**Figure 1:**
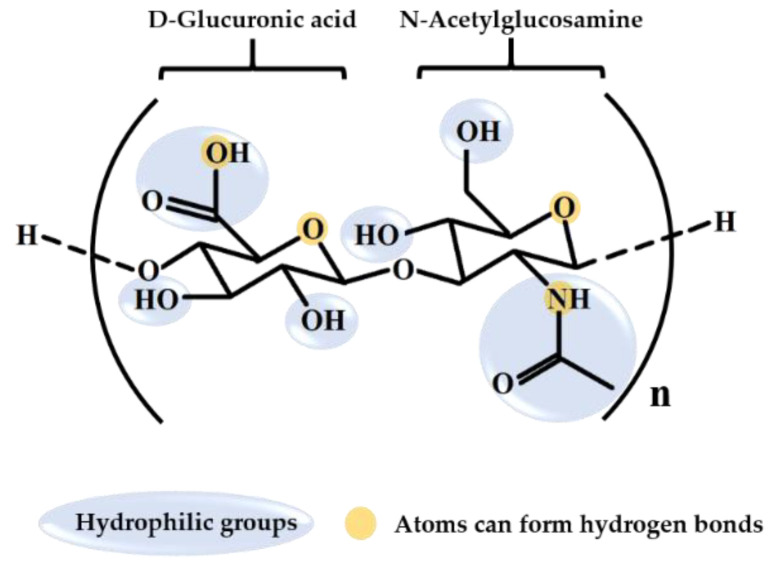
Chemical structure of hyaluronic acid.

Hyaluronic acid (HA) is a natural high-molecular-weight biopolymer. It belongs to the group of long linear nonsulfated glycosaminoglycans (GAGs) with repeating disaccharide units of glucuronic acid and acetylglucosamine [1,2,3] (Figure 1). HA contains multiple hydrophilic functional groups, including carboxyl, hydroxyl, and acetamido groups [4,5]. The abundant hydroxyl groups form a hydrogen bond with water, leading to a high capacity to retain water in a spiral chain [6]. HA’s biocompatibility is because of its high structural homology with endogenous HA in humans [7]. When dissolved in aqueous solution, HA swells and the chains of HA entangle and become random coils, leading to its unique viscoelasticity [6,8,9]. Because of its advantageous characteristics, such as high water retention, high biocompatibility, and viscoelasticity [10], HA has a wide range of applications for medicine, aesthetic medicine, and cosmetics.

Various biological functions of HA are related to its level of polymerization. High-molecular-weight HA (≥10^6^ Da) inhibits inflammation through its interaction with the cluster of differentiation 44 (CD44) cell surface receptor and the HA-mediated motility receptor [4,11]. High-molecular-weight HA can also form a film on the skin’s surface that retains water and prevents water loss [1,12]. Conversely, low-molecular-weight HA (<10^6^ Da) promotes the macrophage inflammatory process by macrophage activation to remove infectious materials from the wound site [4,13]. Low-molecular-weight HA has a high capacity to penetrate skin that ensures a strong moisturizing ability [1,12]. Reducing the molecular weight of high-molecular-weight HA through physical or chemical methods can increase HA’s potential applications. Physical pretreatment methods, such as ultrasonic degradation [14,15], ozone treatment [16], electron beam [17], gamma ray [13] or microwave irradiation, and thermal treatment [17], do not destroy the chemical structure of HA. However, chemical methods such as enzymatic [18] and acid degradation [15] break the structure of HA.

HA is synthesized in humans by HA synthases, including hyaluronan synthase 1, hyaluronan synthase 2, and hyaluronan synthase 3, mostly present in the extracellular matrix of vertebrate tissue [4,5,19]. HA is present in the umbilical cord (4100 μg/g), synovial fluid (1400–3600 μg/g), dermis (200 μg/g), vitreous body (140–338 μg/g), and brain (35–115 μg/g) [20,21,22,23]. HA was originally extracted from animal tissue [4,24,25], but the process is tedious and complicated and the quality uncontrollable, with potentially low yield and a risk of protein and virus pollution [6,26]. Microbial fermentation has been widely used to produce HA but the safety and compatibility with the human body of such HA is of the utmost concern [4,6,24,25,26]. Numerous toxicity assays of HA have demonstrated that in terms of cytotoxicity, L929 mouse fibroblast cells have no toxic effect [7,27,28,29]. The carcinogenicity of HA was tested by the mouse, and no tumor growth was observed after delivered through oral administration of 200 mg/day for 4 weeks [7,30,31]. Concentrations of 670 mg/kg/day HA in rats and 50 mg/kg/day HA in rats and rabbits delivered through oral administration and subcutaneous administration have exhibited no adverse effects in reproductive and developmental toxicity assays [7,31,32,33]. These results have demonstrated the safety of HA.

HA exhibits good versatility in application. Because it retains water well, it can be employed to increase tear film stability to treat dry eye disease [34]; it can be added to contact lens during production [35] and contact lens care solutions [36] to increase comfort; and it can be used in skin care products [37] or added to health food to reduce dry skin [38,39]. Due to its high biocompatibility, HA can enhance tissue growth to heal wounds when combined with receptors on the cell surface [13] or form dermal fillers to improve wrinkles [40]. HA’s high viscoelasticity can decrease friction on the ocular surface during blinking to treat dry eye [34] and keratoconjunctivitis sicca [41] or improve synovial fluid quality and reduce joint friction in the treatment of osteoarthritis [42].

## 2. HA Identification and Quantification Methods

Despite its already numerous applications in various fields, it is necessary to properly inspect and confirm HA’s physical properties, such as its structure type, molecular weight, and concentration, to determine its ideal use. HA extracted from animals and microorganisms alike requires multiple purification steps [14,43,44,45]. The identification and quantification of purified HA requires various pretreatments to prepare HA for further analysis, including enzymatic degradation [18,46,47], acid hydrolysis [48], labeling [48], acid digestion [49], and derivatization [46,48].

After pretreatment, the HA can be detected through traditional electrophoresis of a membrane [50] or gel [18,44,47] matrix. Compared with membrane or gel electrophoresis, advanced capillary electrophoresis is simple, rapid, and sensitive, with high separation efficiency [51,52,53]. Scientists use high-performance liquid chromatography (HPLC) with ultraviolet or mass detection to analyze HA in complicated biological samples [48,54,55]. Although HPLC columns are expensive, this technique is effective for analyzing HA quantitatively and qualitatively with high detection sensitivity. Other analytical techniques applied to HA analysis include Fourier transform infrared spectroscopy [15,16,17], circular dichroism [15], ultraviolet–visible absorption spectroscopy [15,16,17], and nuclear magnetic resonance spectroscopy [13,16,56]. Quantitative and qualitative methods of analyzing HA are summarized in Table 1.

Agarose gel electrophoresis [44], HPLC with a refractive index detector [13,14,43], or a multi-angle light scattering detector [15,47,51] can be applied to identify the molecular weight of HA after extraction from animals, microbial production, or degradation. To reconfirm HA’s molecular weight, HPLC with a viscometer detector and multi-angle light scattering detector can used to detect changes in HA’s viscosity and molecular radius [14]. These methods can be used to compare the antioxidant [14,43] and antiglycation activities [14] of HA of various molecular weights, identify HA in mouse plasma [51], and prepare low-molecular-weight HA [15,47] to promote skin wound healing [13].

HA quantitation can be performed through a carbazole method [44,56], enzyme-linked immunosorbent assay [50], capillary electrophoresis [51,52,53], or HPLC, coupled with triple quadrupole mass spectrometry [48], fluorescence [46], mass spectrometric detector [49,54], or an ultraviolet–visible detector [55], to determine the quantification and detection limit [46,48,49,54,55]. The detection limit of capillary electrophoresis is 1-15 ppm [51,52,53]. The detection limit of HPLC coupled with fluorescence or different mass spectrometry is 2.7 ng [46] and 0.11-4 ppm [48,49]. HPLC with an ultraviolet–visible spectrum reached a detection limit of 0.45 ppm [55]. These methods can also be applied to verify the labeled and declared content of HA [46], quantitatively compare HA in edible fish intestines and liver [48], quickly quantify HA in biological and cosmetic products [52], quantify HA in contact lens multipurpose solution, and analyze the release behavior from contact lenses [55]. HA is also used in highly diverse and complex samples; thus, developing improved methods of sensitivity detection is vital. Although each method has its unique purpose, current HA analysis requires simple sample preparation, swift data acquisition, and high sensitivity.

## 3. Applications of HA in Ophthalmology

HA is found in various tissues in the eye, including the aqueous humor, trabecular meshwork, and vitreous body; through the cell surface glycoprotein CD44, HA can bind easily with the cell membrane [57]. HA also moisturizes the eye, increases biocompatibility, and prolongs drug residence time to enhance drug delivery [37,57,58,59,60]. These properties allow for HA’s use in artificial tears, eye drops, in situ forming hydrogels, modified nanoparticles, intravitreal injections, and tissue engineering (Table 2).

### 3.1. HA in Artifical Tears and Eye Drops

Dry eye syndrome (DES) is a common ocular disorder related to age, gender, diet, environment, disease, or surgery or occurring as a medication side effect [61,62,63]. HA can be added to artificial tears to enhance and extend the duration of moisture retention and therefore alleviate DES [60,64,65,66,67]. In eye drops, a high content of HA stabilizes tear films and increases conjunctival goblet cells [68]. Along with HA concentration, Kojima and colleagues reported that high-molecular-weight HA in eye drops exerts anti-inflammatory effects [69]. Hybridization of high and low-molecular-weight HA in eye drops can protect against dehydration of the corneal cell and promote wound healing [70]. A clinical study indicated that high-molecular-weight HA eye drops can be an alternative treatment for patients with severe dry eye [64].

HA can take different forms in eye drops and can be used in combination with another compound, act as a vehicle for drug delivery, or form a bifunctional peptide polymer. HA can be used with triglycerides, phospholipids, vitamin B12, coenzyme Q10, hydroxypropyl guar, antibiotics, or steroids. HA used with above compound could increase tear film thickness [71], improve oxidative stress in the conjunctival epithelium of patients with dry eye [72], sustains ocular surface [73,74], and reduce DES symptoms [67,75,76]. Eye drops containing HA are effective DES pharmaceutical vehicles [77]. HA can be combined with other compounds or peptides to heal wounds [41] and to sustain ocular surface lubrication [78].

### 3.2. In Situ Forming Hydrogel

Ointment is a more viscous topical treatment than eye drops and increases the residence time on the ocular surface to enhance drug absorption, although blurred vision is an unwelcome side effect [79]. In situ gel has the advantages of both an aqueous solution and an ointment. In situ gel is thermosensitive; it is an aqueous solution at low temperature and, as temperature rises, becomes gelatinous. High viscosity prolongs ocular residence time, which is a convenient property for ophthalmologic use [79,80,81]. HA is a natural polysaccharide with viscous properties, and it can be used to adjust in situ gel viscosity and degradation time [57,79,80,81,82,83].

Fungal keratitis, a type of cornea infection caused by a fungus, can lead to blindness [84]. Hydrophobic ketoconazole is an effective treatment for fungal keratitis, but has low solubility in aqueous solutions. Zhu and coworkers developed an in situ gel utilizing poly(N-isopropylacrylamide) and HA as a vehicle for ketoconazole. To improve biocompatibility and ocular surface residence time, in situ gel with HA was used to prolong drug release, with no irritant reaction exhibited in the rabbit eye tests. As well as creating a more viscous gel, HA can increase the lower critical solution temperature to body temperature for ease of use [79]. The sol–gel temperature also depends on HA concentration [83]. Researchers have demonstrated that in situ gel aids drug absorption and drug delivery [80,81] and improves eye comfort [82].

### 3.3. HA-Modified Nanoparticles

Using nanoparticles (NPs) is a practicable method to increase drug absorption. NPs can be used as drug carriers for hydrophobic or unstable drugs. NPs interact with the corneal epithelium and enter corneal cells to decrease drug degradation [57,85]. After modification by HA, the use of NPs leverages such advantages as improved lubrication, long ocular residence time, and enhanced drug absorption [85,86]. Additionally, HA is prone to bind with the cellular receptor CD44, rendering HA–NPs a suitable vector for gene therapy [57].

Huang and coworkers used HA to modify gelatin loaded with epigallocatechin gallate (GEH), a green tea polyphenol that can decrease inflammation on the ocular surface [87]. The modified gelatin NPs were added to eye drops to improve DES in rabbits. HA increases the concentration of NPs on the cornea to enhance drug absorption. Following 3 weeks of topical administration, clinical tests indicated that GEH reduced inflammatory cytokines (tumor necrosis factor alpha, interleukin 6, interleukin 1 beta and interleukin 8) in the cornea and improved dry eye symptoms [85].

An HA–NP eye drop for glaucoma can achieve the drug absorption effect too. Patients with glaucoma experience high intraocular pressure leading to optic atrophy, whereby peripheral vision is gradually lost, eventually resulting in blindness [88,89]. Treatment for glaucoma has low bioavailability and drug retention time, with more frequent administration leading to adverse effects. Wadhwa and colleagues used HA-modified chitosan (a biodegradable polysaccharide) nanoparticles (CS–HA–NPs) loaded with medicine to improve drug retention time and reduce side effects from frequent administration [89].

Poly(lactic-co-glycolic) acid NPs coated with HA were applied to encapsulate lutein to treat age-related macular degeneration [90]. Because the macula contains high concentrations of lutein to protect photoreceptor and retinal pigment epithelium cells, properly suppling lutein prevents this degeneration [91]. Lutein is hydrophobic and easily degraded by light and heat, but NPs can enhance its bioavailability, physical stability, and decrease degradation. Furthermore, after modification of NPs with HA, HA–NPs can deliver lutein without difficulty due to binding with CD44 [90].

Nanomicelle is a surfactant that can easily encapsulate hydrophobic drug that cannot formulate in solution, but the toxicity and irritation to eye structure are disadvantages [92,93]. HA can prolong the drug duration time and increase the bioavailability to protect cornea from the toxicity and irritation caused by surfactant. Terreni, E. and coworkers used HA to modified nanomicelle to prolong drug resident time and decrease surfactant toxicity [94].

### 3.4. HA Application in Intravitreal Injections

Intravitreal injections can be used for the administration of drugs, gene therapy, or the artificial vitreous humor. These injections can break through barriers that affect drug absorption such as corneal tissue, tear flushing, tear secretions, or drug delivery to target area located in the posterior eye [95,96]. Disadvantages of intravitreal injection include an increase of intraocular pressure and the necessity of repeated injections due to rapid drug release [96,97]. These drawbacks are burdens on the patient and may induce complications [98,99,100]. The high biocompatibility, biodegradability, and capacity to prolong drug release of HA make it ideal as an artificial vitreous material or intravitreal injection drug component.

Drug intravitreal injections are widely used in ophthalmology, but the short-lived effect of the drugs results in a need for repeated injections, which can cause side effects. Yu and coworkers encapsulated bevacizumab, a treatment for ocular neovascularization, into a hydrogel mixed with HA and dextran to slow drug release. The results demonstrated that the gel was highly biocompatible, facilitated a more stable release of drugs compared with a bolus injection, and could sustain drug effects for more than 6 months [99]. NPs can also be administered through intravitreal injection [101,102,103]. NPs protect drugs from degradation, extend the drug releasing duration, and reduce the frequency with which injections must be administered. After modification with HA, NPs can effectively combine with the affected cell through the binding of HA and CD44. Connexins, a 43-kDa mimetic peptide, was loaded into HA–NPs to treat retinal disease, and their injection into Wistar rats’ injured eyes improved drug duration and cell uptake [102].

Gene therapy uses intravitreal injection to break through barriers and reach the target area [95]. The eye is suitable for gene therapy because of its immune privilege—the inflammatory immune response is avoided to preserve its function [104,105]. Martens and coworkers used cationic *N,N’*-cystaminebisacrylamide-4-aminobutanol modified with HA as a vehicle loaded with anionic plasmid DNA for gene therapy. HA enhanced the connection between vehicle and target cells (ARPE-19) by binding to CD44. The researchers reported that low-molecular-weight HA can enhance the intravitreal injection of gene therapy [95].

HA is a vitreous humor component and can therefore be used for artificial vitreous humor [106,107]. HA can replace the silicone oil in vitrectomy and prevent side effects, cytotoxicity, silicone oil emulsification, or second surgery because of its biocompatibility and biodegradability [108]. Raia and colleagues combined HA and silk fibroin through enzymatic crosslinking, using horseradish peroxidase and hydrogen peroxide to increase stability. Their study demonstrated that silk–HA hydrogel possesses biocompatibility and stability as a vitreous substitute [106].

### 3.5. Tissue Engineering

HA is a critical component in the extracellular matrix. The extracellular matrix assists in cell migration, adhesion, and the proliferation [109,110] of such cells as adipose-derived stem cells and limbal epithelial stem cells [111,112]. These cells aid in corneal regeneration; specifically, by making a tissue-adhesive scaffold, corneal wound healing can be promoted [113]. Because of HA’s capacity for promoting cell growth and wound healing, Liu and coworkers crosslinked collagen and gelatin with HA to fabricate a film. An optimized ratio of HA, collagen, and gelatin has better tensile strength, elongation strength, water absorption, and contact angle. The morphology and methylthiazol tetrazolium assay demonstrated the growth and proliferation of human corneal epithelium cells, demonstrating that collagen–gelatin–HA (ratio 6:3:1) has appropriate biocompatibility for tissue engineering [110]. HA hydrogel can also be used in corneal tissue engineering. Overrun-processed porous HA hydrogels act as cell carriers for corneal endothelium cells and can reconstruct the corneal endothelium. HA hydrogels exhibit biocompatibility with rabbit corneal endothelium cells. In in vivo transplant tests, HA hydrogels were implanted into the anterior chamber of an injured rabbit eye. The results indicated a significant increase in endothelium cells after 4 weeks of treatment. The slit-lamp biomicroscopic image also revealed a transparent cornea [114], indicating that the HA hydrogel had successfully reconstructed the injured cornea. These results demonstrate that HA can be a scaffold for tissue engineering.

**Table 2:** Applications of HA in Ophthalmology.

Ophthalmology Application	Target	HA Function
Artificial tear and eye drop	Ocular surface	1. Increase the moisture retention [60,64,65,66,67] 2. Better tear film stability, ocular surface regularity, and quantity of conjunctival goblet cells [68] 3. Anti-inflammatory effect [64,69] 4. Protect corneal cell dehydration [70] 5. Increase tear film thickness [71] 6. Improve dry eye patients’ conjunctival epithelium oxidative stress [72] 7. Have more effective treatment [73] 8. To reduce the DES symptom [67,75,76] 9. As DES pharmaceutical vehicle [77] 10. Heal wound [41,70] 11. Sustain ocular surface lubricated [74,78]
In situ gel	Ocular surface	1. Adjust the viscosity and degradation time [57,79,80,81,82,83] 2. Increase the lower critical solution temperature for thermosensitive in situ gel [79] 3. Help the drug absorption and drug delivery [80,81] 4. Provide better eye comfort [82]
Nanoparticles	Ocular surface and Retinal	1. Better lubricating, ocular residence time, and drug absorption [85,86,89,90] 2. Increase mucoadhesion [89] 3. Increase cellular targeting by CD44 [57,89,90] 4. Decrease surfactant toxicity [94]
Intravitreal injection	Vitreous humor	1. Increase cellular targeting by CD44 [95,102] 2. Biocompatibility and biodegradable for vitreous humor substitute [100,106,107,108]
Tissue engineering	Corneal	1. Benefit of cell growth and wound healing [110,114]

Abbreviations: dry eye syndrome (DES), cluster of differentiation 44 (CD44).

## 4. Applications of HA on Contact Lenses

HA is biocompatible and effective at retaining moisture on contact lens. HA has been applied in the contact lens field for almost 20 years, and the applications of HA include incorporation into the lenses, surface modification, multipurpose solution supplementation, stabilization of eye medication, and drug release sustainment (Table 3).

### 4.1. HA-Modified Contact Lenses Exhibit Enhanced Comfort

Contact lenses are divided into two classes based on their material, namely, hydrogel and silicon hydrogel lenses. Silicon hydrogel contact lenses have better oxygen permeability, but their hydrophobic characteristics may irritate eyes. When contact lenses soaked in multipurpose solution absorb HA, HA can decrease the surface roughness of the lens [115]. HA-modified contact lenses exhibit enhanced surface water retention [116], reduce protein adsorption [117,118], and slow tear removal, which considerably increases comfort. Moreover, the surfaces of contact lenses are coated with HA to enhance its biocompatibility with human corneal epithelial cells [119].

### 4.2. HA Adsorption and Desorption on Contact Lenses

Because wearing contact lenses for prolonged periods may cause dry eye, HA-soaked contact lenses retain moisture, thus solving this problem. In our previous study, we demonstrated that contact lenses soaked in a higher-concentration HA solution released more HA, although most HA was rapidly released in the first 30 min. When immersed in the same concentration of HA solution, methafilcon A food and drug administration, (FDA), Group Ⅳ, high water content, ionic polymer) contact lenses demonstrated the highest attachment ability, and the polymacon (FDA Group Ⅰ, low water content, nonionic polymer) demonstrated the slowest release rate [55]. Scheuer and colleagues revealed that soaking lenses in multipurpose solution containing HA overnight can promote retention of HA on the lenses and that different contact lens materials that include hydrogel (four group, including low/high water content and nonionic/ionic polymer) and silicon hydrogel have different HA release rates [120]. Absorbed HA on contact lenses is typically released in the first 2–3 h [121,122].

### 4.3. HA Release on Contact Lenses

The treatment of ocular diseases usually involves the administration of eye drop medicine to the anterior eye, but this method requires application several times a day and is burdensome to older patients or those with chronic disease. According to researchers, approximately 5% of drugs reach the cornea through this administration method [7]. Because contact lenses are widely used, convenient, and inexpensive, they can act as the medical delivery vehicle of continuous-release drugs. The incorporation of HA into contact lenses not only aids the treatment of dry eye but also serves as a wetting or comfort agent.

HA is the foremost molecule in the treatment of dry eye through contact lenses. The release rate of HA on HA-soaked contact lens is rapid in the first 6 h and then slows [123]. HA incorporated into contact lenses can be continuously released for 48 h [124]. Although a higher HA concentration has a relatively high initial release percentage, an increase in HA concentration does not extend release time duration [123]. For HA-laden contact lenses that entrap HA to prolong release duration [125], the HA release time was up to 15 days in a rabbit tear fluid test [123]. A molecular imprinting technique controls the release of HA from hydrogel contact lenses at the rate of 6 μg/h for 24 h [126].

In regards to HA as a wetting or comfort agent applicate to contact lenses, Weeks and coworkers reported that HA incorporated into hydrogel and silicone hydrogel contact lenses continuously released HA for at least 3 weeks [127]. HA or drugs can be implanted into contact lenses to avoid changing lens properties. HA is used as a comfort agent with various antibiotic and glaucoma drug treatments to make patient wear more comfortable. Contact lenses implanted with HA can sustain the release of HA up to 96 h to achieve healing effects [128,129,130].

### 4.4. Application of HA in Medical Lenses

HA assists in controlled drug release on contact lenses. Nguyen and coworkers fashioned contact lens discs containing HA and drugs. Contact lenses containing HA incorporate and release more of the drug, especially hydrophobic drugs, for 6 days [131]. HA is also used as an additive to hold timolol, a drug for the treatment of glaucoma. When silicon hydrogel contact lenses were loaded with HA, the drug quantity on the lens increased as did the release by approximately 2 days [132]. HA can be prepared as a film to coat contact lenses and to temporarily adhere to cells, whose viability can then be maintained to repair corneal damage [133].

**Table 3:** Applications of HA on Contact Lens.

Application	HA Attach Contact Lens Manner	Release Time	Other Drugs
Comfortability	Surface modification	-	-
Moisturization	Immerse in HA-contain solution	-	-
molecule of treatment dry eye	Immerse contact lens in HA-contain drug solution or incorporate in contact lens	24 h (6 μg/h) [126], 48 h [124] and 96 h [125], 15 days [123]	-
Wetting or comfort agent		96 h [128] and at least 3 weeks [127]	Timolol [128,130] Bimatoprost [130]
Drug release control		2 [132] and 6 [131] days	Ciprofloxacin-HCl and dexamethasone phosphate [131] Timolol [132]
Cell adhesion for corneal damage repair	HA-contain film coating in contact lens inner surface	-	-

## 5. Conclusions

HA reveals outstanding properties of hydrophilic, safety, compatibility, and special viscoelasticity. It has been widely used in the field of ophthalmology. The applications of HA in artificial tear and eye drop, in situ gel, nanoparticles, intravitreal injection, and tissue engineering enhance eye comfortable and cure eye diseases. Moreover, HA applied in contact lenses can improve wearing comfort, control drug release, and even be the molecule to treat eye-related disease. Because HA is a macromolecule with flexible molecular weight, manufacturing specific sizes of HA molecules may enhance the stability in the versatile application. In the future, chemical medication of HA can further change the properties to adapt the applications in the medical field. More importantly, HA is also a biodegradable material, which can be used without increasing the burden to the environment.

## Figures and Tables

**Table 1:** Methods of analysis applied to hyaluronic acid (HA).

Analysis Method	Hyaluronic Acid Characterization	Sample	Linear Range	LOD	Detection Range
FTIR	1. 3412–3435 cm^−1^ (O-H and N-H stretching) 2. 2916-2919 cm^−1^ (C-H stretching) 3. 1632–1653 cm^−1^ (amide I), 1553–1563 cm^−1^ (amide II), 1320 cm^−1^ (amide III) 4. 1617 cm^−1^ (asymmetric C=O stretching) 5. 1411–1416 cm^−1^ (symmetric C-O stretching) 6. 1150 cm^−1^ (O-bridge of C-O-C group), 1079 cm^−1^ (C-O, C-C groups), 1042 cm^−1^ (C-OH group) [15,16,17]	2 mg [44,45]	-	-	-
CD	1. 183 nm (carboxyl π–π* transition). 2. 187 nm (π–π* transition of GlcNAc) 3. 210 nm (π–π* transition carboxyl group) [15]	0.5 mg/mL [15]	-	-	-
UV-Vis	1. ~210 nm (carbonyl-or carboxyl groups) [43] 2. 265 nm (double bond) [13,17]	0.5–2 mg/mL [13,15]	-	-	-
NMR	1. 25, 57, 63, 71, 76, 79, 83, 85, 103, 106, 177 ppm (^13^C) [45] 2. 171 ppm (carboxylate carbon), 175 ppm (acetamido carbonyl carbon (^13^C) [13] 3. 1.89 (CH_3_-group), 3.70 (CH_2_-group), 3.69 (NH-group), 4.3–4.4 (OH-group) ppm (^1^H) [56]	-	-	-	-
Carbazole	516 [56] or 540 nm [44]	-	-	-	0.03–1.7 g/L [44] or 6–10 g/L [56]
ELISA	450 nm [50]	-	-	-	150–250 ng⁄µg [50]
Gel EP	1. 515 nm (ANTS-label) [18] 2. Alcian blue with silver and Stains-all stain [44,47]	-	~25–500 kDa [44,47]	-	4–20-mer [18]
Membrane EP	Polysaccharides in Alcian blue stain [50]	-	-	-	-
CE	200 nm [51,53], 195 nm [52]	-	50–150 ppm [52] or 0.02–3.0 ppm [53]	1 ppm [52] 9 ppm [53]	-
HPLC coupled with MALS	-	0.05–0.1 mg [14,47]	-	-	75–1000 kDa [15] or 510 kDa [51]
HPLC coupled with VD	-	0.1 mg [14]	-	-	470–1600 (mL/g) [14]
HPLC coupled with RID	-	0.1–2 mg [13,14,43]	270–2000 kDa [43]	-	60–23,000 kDa [13] or 180–1100 kDa [14]
HPLC coupled with FL	λex = 428 nm, λem = 525 nm [46]	-	1.6–47 μg [46]	2.7 ng [46]	-
HPLC coupled with MS	Positive ionization mode [48,49] Negative ionization mode [46,54]	-	0.5–500 pmol [54] or 0.01–1.0 mg/mL [48]	0.6 g/mL [49] 0.1 ppm [48]	-
HPLC coupled with UV	205 nm [55]	-	0.01–0.15 mg/mL [55]	0.45 ppm [55]	-

Analysis instrument abbreviations: Fourier transform infrared spectroscopy (FTIR), circular dichroism (CD), ultraviolet–visible absorption spectrum (UV–Vis), nuclear magnetic resonance spectroscopy (NMR), enzyme-linked immunosorbent assay (ELISA), electrophoresis (EP), 8-aminonaphthalene-1,3,6-trisulfonic acid (ANTS), capillary electrophoresis (CE), high-performance liquid chromatography (HPLC), multi-angle light scattering (MALS), viscometer detector (VD), refractive index detector (RID), fluorescence (FL), excitation wavelength (λex), emission wavelength (λem), and mass spectrometry (MS). The limit of detection abbreviation is LOD.

## Data Availability

Not applicable.
